# Wave discrimination at C-band frequencies in microstrip structures inspired by electromagnetically induced transparency

**DOI:** 10.1038/s41598-021-82618-1

**Published:** 2021-02-03

**Authors:** Abdul Jabbar, Rashad Ramzan, Omar Siddiqui, Muhammad Amin, Farooq A. Tahir

**Affiliations:** 1 Research Institute for Microwave and Millimeter-Wave Studies, National University of Sciences and Technology, Islamabad, Pakistan; 2 National University of Computer and Emerging Sciences, Islamabad, Pakistan; 3 College of Engineering, Taibah University, Madinah, Saudi Arabia

**Keywords:** Engineering, Electrical and electronic engineering

## Abstract

We present the design and practical implementation of a microstrip diplexer based on the wave discrimination property associated with the electromagnetically induced transparency (EIT)-like effect. The EIT is a quantum interference phenomenon which happens between two atomic transition pathways and allows wave propagation within a medium’s absorption spectrum. Here, we exploit an analogous interference mechanism in a three-port microstrip structure to demonstrate a diplexer based on the EIT-like effect in the microwave regime. Since the transparency is accompanied by a high transmission and strong dispersion characteristics, compact frequency discriminating structures that can resolve nearby frequencies with high isolation can be devised. Our proposed C-band diplexer consists of pairs of unequal open-circuit stubs, which resonate at detuned frequencies and interfere to form the EIT-like passbands for diplexer action. The design is highly compact and scalable in frequency for both PCB and on-chip applications. A prototype of diplexer is fabricated for the center frequencies of lower and upper passbands at 4.6 GHz and 5.5 GHz respectively. The transmission zeros are designed at the complementary channels so that the two passbands are highly isolated presenting the isolation of about 40 dB. The measured insertion loss of lower and upper passband is 0.59 dB and 0.61 dB respectively. Measured input return loss is better than − 15 dB, while the output return losses are well below − 12 dB. Moreover, a decent value of about 200 is achieved for the group refractive index around the EIT-like passbands, which reveals the slow wave characteristics of the proposed EIT-based diplexer.

## Introduction

The term electromagnetically induced transparency (EIT) was first coined by Harris et al. in 1990^1^ when they showed the possibility of wave propagation in an optically opaque medium by means of detuned laser interference. Boller et al.^2^ performed a subsequent experiment in the Strontium vapor to practically demonstrate the formation of a transparency window within the Lorentz absorption region. The researchers’ captivation towards the EIT was not only due to the fact that it rendered an otherwise opaque medium transparent, rather the transparency effect itself can be replicated at any other frequency by ubiquitous wave interference phenomenon. More intriguing was the steep linear dispersion profile that accompanied the transparency^3,4^. The rapidly changing phase spectrum gave rise to yet another series of fascinating phenomena such as efficient wave mixing^5^, extremely slow group velocities (up to 8 m/s)^6^, and the stopped light concept^7^. Inspired by the EIT’s sharp spectral features, several studies followed the quantum approach of resonant wave mixing to reproduce the EIT-like dispersion in optical structures. Optical routing based on light storage property of the EIT effect has been demonstrated for practical applications in quantum information and all-optical network^8,9^, while coherent interference of light was manipulated to obtain the EIT-like dispersion in on-chip micron-size silicon optical resonators^10^. On the similar lines, the EIT-like response was realized by optical resonator coupling such as with ring resonators^11^ and by the coherent interference of photonic crystal cavities^12^. From the practical aspect, the rapidly varying phase features arising from the resonant coupling have been exploited to design novel EIT-inspired applications. The slow wave applications originate from the fact that the group velocity in a medium depends on the frequency derivative of the phase slope. The steeper and linear slope leads to uniform and slower group velocity across a bandwidth^13,14^. Slowing the light to the extent of freezing has led to the applications which can revolutionize the future of computing including quantum memories^15–18^ and optical buffers^19^. Another area of applications is based on the sharp amplitude response that is obtained when spectrally nearby resonances are coupled to form the EIT within a narrow band. The high-Q resonant devices find applications in highly selective optical filters^20^, and sensors with increased sensitivities^21,22^.

While most of the research on the EIT-like effects has been done in the optical regime, plenty of metamaterial-based designs have been reported in the microwave regime as well, which support the EIT-like effects. These EIT-like responses are investigated to provide a platform for many potential applications such as the realization of long range EIT, all-optical signal processing, light tunneling effect, rapid optical response, slow light applications at room temperature and chiral slow light devices to name but a few^23–39^. Moreover, the EIT-like effects are also investigated and employed at microwave frequencies using planar microstrip based structures and RLC based circuits for different applications such as microwave sensing, switching and phase shifting^40–48^. The EIT-like dispersion in MHz frequencies was obtained by placing two micro-resonators in close vicinity so that their whispering gallery modes coherently interact^41^. The EIT-based sensing was reported in a 3D structure consisted of stacked layers of Jerusalem crosses^42^, and a microstrip guided-wave circuit loaded with detuned magnetic dipoles^48^. In both of these structures, large transmission losses were reported due to weak coherent interaction between the resonant modes. In a similar vein, studies have also been conducted to represent the EIT-like and Fano resonant modes in terms of their equivalent lumped circuit representations^40,43,47^. These equivalent circuits helped to understand the modelling and analogues of the EIT-like effects. Amin et al.^44–46^ proposed a much simpler geometry supporting the EIT-like effects that demonstrated better transmission characteristics than the previously proposed microwave structures^41,42,48^. Their structure consisted of two detuned open-circuit stubs that loaded a microstrip transmission line and provided a simpler way to fabricate compact microwave devices. Since the stubs and the transmission path that guided the EIT-like resonance are well connected, there was a strong wave coherence that led to the high transmission coefficient. These double-stub based EIT-like structures can be applied where highly selective filtering or wave discrimination of spectrally close resonances are desired.

Although the EIT-like response due to the pair of detuned stubs^45,46^ forms the foundational basis of the microstrip-based EIT-like effect, the applications proposed therein were of primitive nature exploiting the dispersion properties of a single EIT-like structure. We strongly feel that the sharp dispersion features associated with the EIT-like phenomena can be exploited to build compact devices in the microwave domain. One of the important microwave components is the *diplexer* which is used for frequency discrimination, channels selection and signal synthesis. To date, several techniques have been applied to design microwave diplexers, such as uniform impedance resonators, stepped impedance stub-loaded resonators, dual mode stub-loaded resonators and quarter-wave resonators^49–54^. Many of these techniques require higher order filters and complex designs to obtain distinct frequency bands for diplexing action. In this paper, we investigated and practically demonstrated a novel diplexer that is based on the concept of the EIT-like effect using much simpler microstrip based geometry.

## Proposed diplexer design

**Figure 1:**
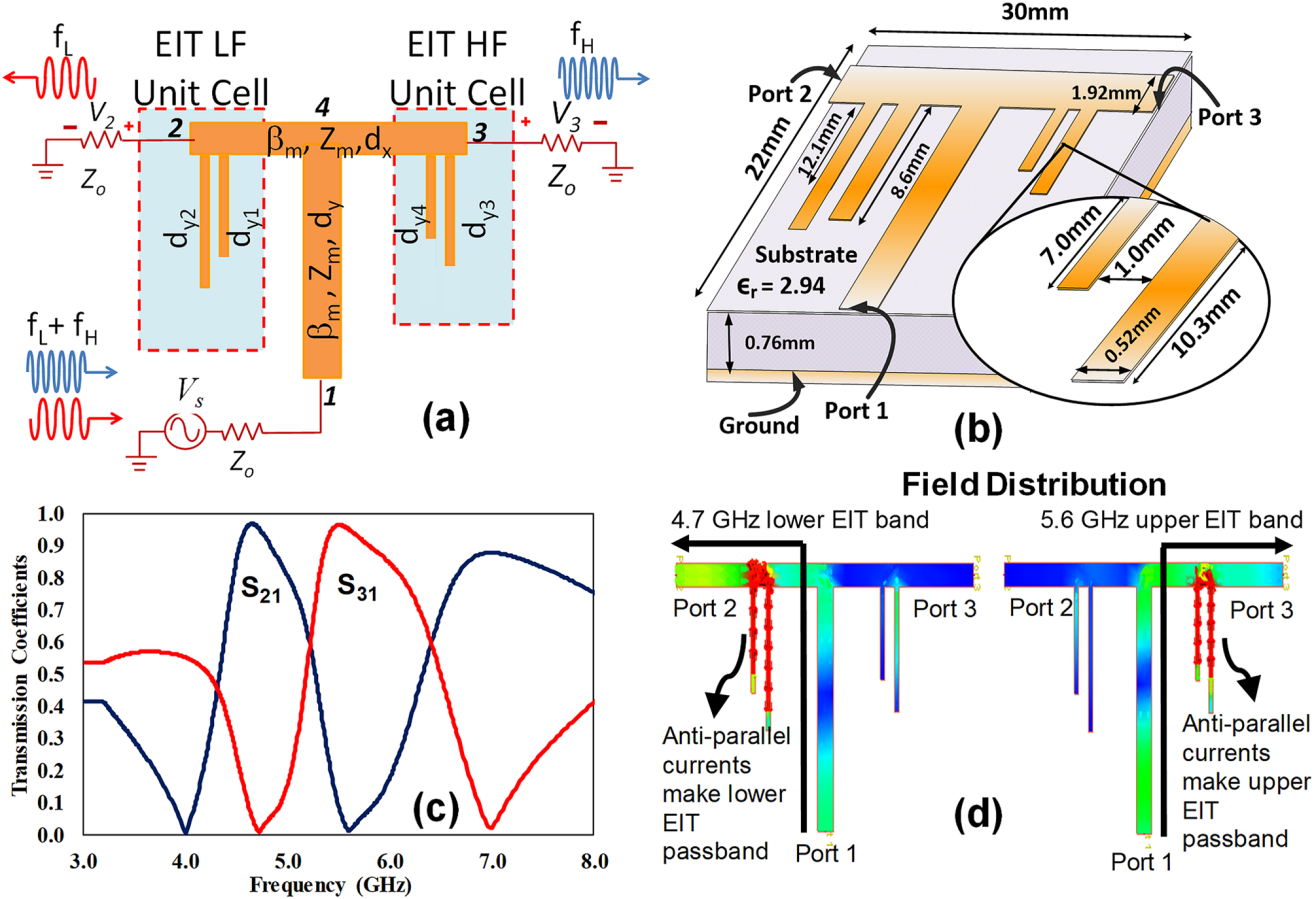
(**a**) Schematic model of the proposed microstrip Diplexer based on the EIT-like effect. The power injected at node 1 is discriminated in frequency such that the 4.6 GHz spectral component appears at port-2 and 5.5 GHz at port-3. Each transmission line segment is modelled by its forward transmission matrix. All the feeding lines have a characteristic impedance of 50 Ω and the stubs 100 Ω (**b**) The diplexer implemented on Rogers 6002 substrate of ε_r_ = 2.94. (**c**) Transmission coefficients s21 and s31 show the passbands at the design frequencies. The high isolation can be seen by recognizing the transmission null of one channel occurring at the passband of the other. (**d**) Numerical simulations of current distributions performed in Agilent’s Momentum full-wave simulator in the two EIT-like passbands. The two stubs in the excited EIT-like structure show anti-parallel currents indicating the destructive interference.

The principle of proposed diplexer and the related circuit analysis is illustrated in Fig. 1. The wave discrimination (diplexing action) is achieved by connecting two pair of stubs with the main energy thread (the transmission line) towards each output port, as shown in Fig. 1a. Each output side consists of a pair of open-circuit stubs supporting a separate transparent band so that two spectrally adjacent frequency channels appear at port-2 and port-3. The EIT-like effect in the proposed structure is introduced by the resonance detuning effect. The resonance detuning is often necessary to control the required spectral interference to generate the EIT-like response. Our proposed design is based on two coupled detuned stubs. The additional stub connected to the main transmission line in series leads to the geometrical asymmetry that stems from the path difference (junction length) between the two stubs relative to the input port as shown in Fig. 7a in the later section. The junction length adds to the path of the secondary stub, therefore effectively increases its electrical length. In this context, for the EIT-like response to be produced, the primary stub closer to the input port works as a bright mode (d_y1_ and d_y4_) whereas the detuned resonance in the adjacent secondary stub acts as dark mode (d_y2_ and d_y3_). As a result, the underlying resonance mechanism in the stub configuration undergoes strong mutual interference generated between the resonant electric fields of the slightly displaced adjacent stubs. Therefore, the two detuned stubs in proximity can be approximated as two slightly detuned resonators leading to a narrow band EIT-like response. In the absence of the junction length (path difference), both stubs will exactly overlap, and the EIT-like window will disappear, leading to a Lorentzian profile with a single transmission null at the resonance frequency of the stub.

The diplexer structure (Fig. 1a) can be analyzed by applying the forward transmission matrix (FTM) method^49^. To construct the node solution, the currents propagating away from the four voltage nodes are calculated by using the ABCD matrices. A subsequent application of the Kirchhoff’s current rule results in four simultaneous s-parameter equations, which can be conveniently written in the form of the following F-matrix system:

1 $$ \left[ {\begin{array}{*{20}c} {\begin{array}{*{20}c} {S_{11} + 1} \\ {S_{21} } \\ \end{array} } \\ {S_{31} } \\ {S_{41} } \\ \end{array} } \right] = 2\left[ {\begin{array}{*{20}c} {\begin{array}{*{20}c} {F_{11} } \\ 0 \\ \end{array} } \\ 0 \\ {F_{41} } \\ \end{array} \begin{array}{*{20}c} {\begin{array}{*{20}c} 0 \\ {F_{22} } \\ \end{array} } \\ 0 \\ {F_{42} } \\ \end{array} \begin{array}{*{20}c} {\begin{array}{*{20}c} 0 \\ 0 \\ \end{array} } \\ {F_{33} } \\ {F_{43} } \\ \end{array} \begin{array}{*{20}c} {\begin{array}{*{20}c} {F_{14} } \\ {F_{24} } \\ \end{array} } \\ {F_{34} } \\ {F_{44} } \\ \end{array} } \right]^{ - 1} \left[ {\begin{array}{*{20}c} {\begin{array}{*{20}c} {V_{s} } \\ 0 \\ \end{array} } \\ 0 \\ 0 \\ \end{array} } \right] $$

where the elements of the F-matrix are the coefficients of the Kirchhoff’s current equations and are given in the form of forward transmission (ABCD) parameters of the transmission line segments connected to the related nodes:

2 $$ F_{11} = \frac{{A_{y} }}{{B_{y} }} + \frac{1}{{Z_{o} }} $$

3 $$ F_{22} = \frac{{A_{x} }}{{B_{x} }} + \frac{{C_{y1} }}{{D_{y1} }} + \frac{{C_{y2} }}{{D_{y2} }} + \frac{1}{{Z_{o} }} $$

4 $$ F_{33} = \frac{{A_{x} }}{{B_{x} }} + \frac{{C_{y3} }}{{D_{y3} }} + \frac{{C_{y4} }}{{D_{y4} }} + \frac{1}{{Z_{o} }} $$

5 $$ F_{44} = 2\frac{{A_{x} }}{{B_{x} }} + \frac{{A_{y} }}{{B_{y} }} $$

6 $$ F_{14} = F_{41} = - \frac{1}{{B_{y} }} $$

7 $$ F_{24} = F_{42} = F_{34} = F_{43} = - \frac{1}{{B_{x} }} $$

The ABCD parameters are related to the phase constant (β) and the characteristic impedance (Z) of the transmission lines and are written in the form of the following matrices:

8 $$ \left( {\begin{array}{*{20}c} {A_{yn} } & {B_{yn} } \\ {C_{yn} } & {D_{yn} } \\ \end{array} } \right) = \left( {\begin{array}{*{20}c} {cos\beta_{s} d_{yn} } & {Z_{s} sin\beta_{s} d_{yn} } \\ {Y_{s} sin\beta_{s} d_{yn} } & {cos\beta_{s} d_{yn} } \\ \end{array} } \right) $$

9 $$ \left( {\begin{array}{*{20}c} {A_{k} } & {B_{k} } \\ {C_{k} } & {D_{k} } \\ \end{array} } \right) = \left( {\begin{array}{*{20}c} {cos\beta_{m} d_{k} } & {Z_{m} sin\beta_{m} d_{k} } \\ {Y_{m} sin\beta_{m} d_{k} } & {cosh\beta_{m} d_{k} } \\ \end{array} } \right) $$

where n = 1, 2, 3, or 4 stands for one of the four open-circuit stubs as given in Fig. 1 and k = x or y signifies the x-directed or y-directed transmission line segments connected to the diplexer’s ports. In particular, the phase constant (β_s_ $= \omega \sqrt{{\varepsilon }_{eff}}/c$) is an important parameter as it determines the resonant frequency (f_o_) of the open-circuit stubs. Since each stub resonates at the quarter-wavelength^45^ (${\mathrm{when} \beta }_{s}{d}_{yn}$ = 90°), the approximate length of an open-circuit stub can be calculated using Eq. (10).

10 $$ L_{s} = \frac{c}{{4f_{o} \sqrt {\varepsilon_{eff} } }} $$

The design procedure involves the calculation of the approximate length of each stub from Eq. (10) by considering the fact that the EIT-like passband of each pair of stubs should fall in between the resonances of the two interfering stubs. The approximate model is then substituted in Eq. (1) to Eq. (7) and the frequency responses are further optimized to obtain exact lengths that would satisfy the requirements of the designed spectrum.

As a representative example, a diplexer is designed to discriminate 4.6 GHz and 5.5 GHz as center frequencies. The design is implemented on Rogers 6002 substrate (ε_r_ = 2.94) and is depicted in Fig. 1b. Assuming lossless conditions, the transmission coefficients on the two output ports of the optimized diplexer are calculated using Eq. (1) to Eq. (7) as shown in Fig. 1c. The two diplexing bands are marked by near unity transmission property, which is the hallmark of the EIT phenomenon. High isolation results from the fact that the spectral location of the transparency band coincides with the transmission zero of the other diplexer channel. To gain an insight of the underlying resonance mechanism, the associated surface current distributions are calculated using Agilent’s Momentum full-wave electromagnetic simulator. As depicted in Fig. 1d, the high intensity anti-parallel currents show the presence of the destructive interference, and an effect analogous to the quantum EIT is formed. From the circuit theory viewpoint, two shunt branches have oppositely polarized impedance at the EIT-like frequency, thereby acting as a parallel RLC resonance. The pair of open-circuit stubs hence poses a high impedance to the transmission path forcing the dominant power to flow from port-1 towards port-2 and port-3^45^.

To explain the resonant behaviour of the dual-stub based EIT-like structure, each open-circuit stub can be modelled as a series RLC circuit at resonance. Considering the low-frequency EIT response at port-2 (see Fig. 1c) at 4 GHz, the stub d_y2_ becomes quarter-wavelength long and hence can be approximated as a series RLC resonance. Therefore, as it becomes quarter-wavelength long, it looks like a short circuit to the source and provides the least impedance path to the source causing a transmission null. Similarly, the second null of low-frequency channel at 5.5 GHz occurs when the stub d_y1_ attains an electrical length of a quarter-wavelength leading to the dominant current that flows back to the source causing the transmission zero. At a frequency which is approximately in the middle of the two resonances, the impedance of the two stubs is exactly equal and oppositely polarized such that one is inductive and the other is capacitive. At this point, the low-frequency EIT-like structure behaves like a parallel RLC resonance circuit offering high impedance to the source leading to the EIT-like transmission peak as indicated in Fig. 1c. Alternatively, in terms of electromagnetic fields, the two closely placed open-circuit stubs become out of phase (antiparallel currents) leading to destructive interference between two stubs. This leads to the maximum transfer of energy towards port-2 at 4.6 GHz. The similar phenomenon occurs for the upper-frequency channel towards port-3, making two transmission nulls at 4.6 and 7 GHz along with an EIT-like passband in between at 5.5 GHz.

**Figure 2:**
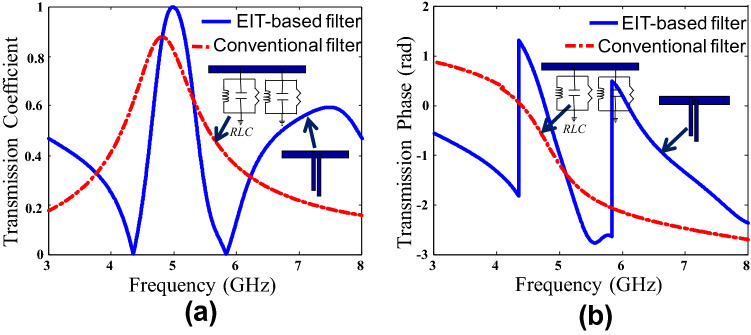
The transmission characteristics of a conventional bandpass filter and an EIT-based filter are analytically calculated using the FTM model (**a**) the amplitude of the transmission coefficient gradually rises for a conventional filter. For an EIT-like structure, the passband is surrounded by transmission zeroes. (**b**) the transmission phase of the EIT-like structure is characterized by sudden changes and phase slope reversals while the phase characteristics change smoothly in case of the conventional filter.

Here it is instructive to highlight the interference phenomenon forming the diplexer frequency response, which is characterized by near unity transmission and high isolation. To show the difference between the regular filtering action and the EIT-like interference, we compare the transmission characteristics of a conventional bandpass filter with those of a low-frequency EIT-like structure (Fig. 2). While the conventional bandpass filter response increases gradually to its maximum value (Fig. 2a), the EIT-like amplitude is characterized by transmission zeroes surrounding the passband. These zeroes are the spectral locations of quarter-wavelength resonances of the individual stubs. More interestingly, the phase of the EIT-like response is marked by steep changes and reversals of slopes (Fig. 2b). This strong dispersion results from the superposition of the electromagnetic fields at some frequencies and cancellation at others. In the absence of any interference, a smooth phase profile would result as shown in Fig. 2b for the conventional filter. It is the strong dispersion that allows the EIT structures to resolve closely located frequencies. Furthermore, the conventional bandpass filter requires several stages to achieve a narrowband response that can resolve close frequencies with reasonable isolation. On the other hand, in the proposed configuration, the frequency response is generated by strong dispersion and the interference action. Hence single-stage EIT-like circuits lead to the desired effects with excellent isolation between the nearby channels.

One of the remarkable features of the EIT-like effect is slow-light. The slowness of a microwave device can be measured in terms of effective group refractive index (${n}_{g}$). The group index can be calculated from the phase of the transmission response using Eq. (11).

11 $$ n_{g} = - \frac{c\partial \varphi }{{L\partial \omega }} $$

**Figure 3:**
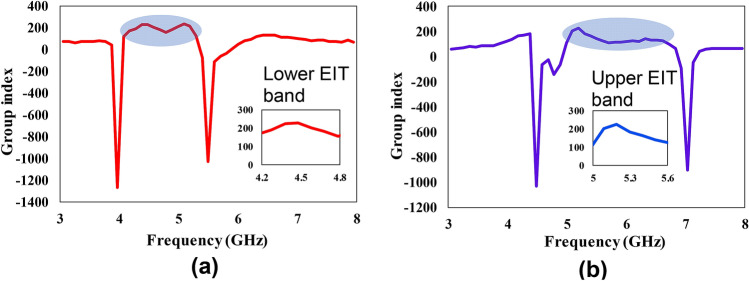
Demonstration of slow wave effect of the proposed EIT-based diplexer in terms of group refractive index. The group refractive index reaches about 200 in the EIT-like bands (**a**) simulated group refractive index for the lower EIT-like band towards port-2. (**b**) the simulated group refractive index for the upper EIT-like band towards port-3.

Here, $c$ is the speed of light, $L$ is the length of the transmission line segment, $\varphi$ is the phase of transmission response and $\omega$ is the frequency. Note that a higher value of group refractive index reveals more slowness of the wave and the reduced group velocity. The group index variation for the two EIT-like bands is shown in Fig. 3. Around the two EIT-like bands (4.6 and 5.5 GHz), the group refractive index exceeds 200 which depicts the slow-wave effect of the proposed structure. Moreover, at the transmission null locations (4 and 5.5 GHz for the lower EIT-like band, 4.6 and 7 GHz for the upper EIT-like band), the group index is negative because of the positive slope of the transmission phase in the vicinity of individual resonances of the stubs.

## Experiment

**Figure 4:**
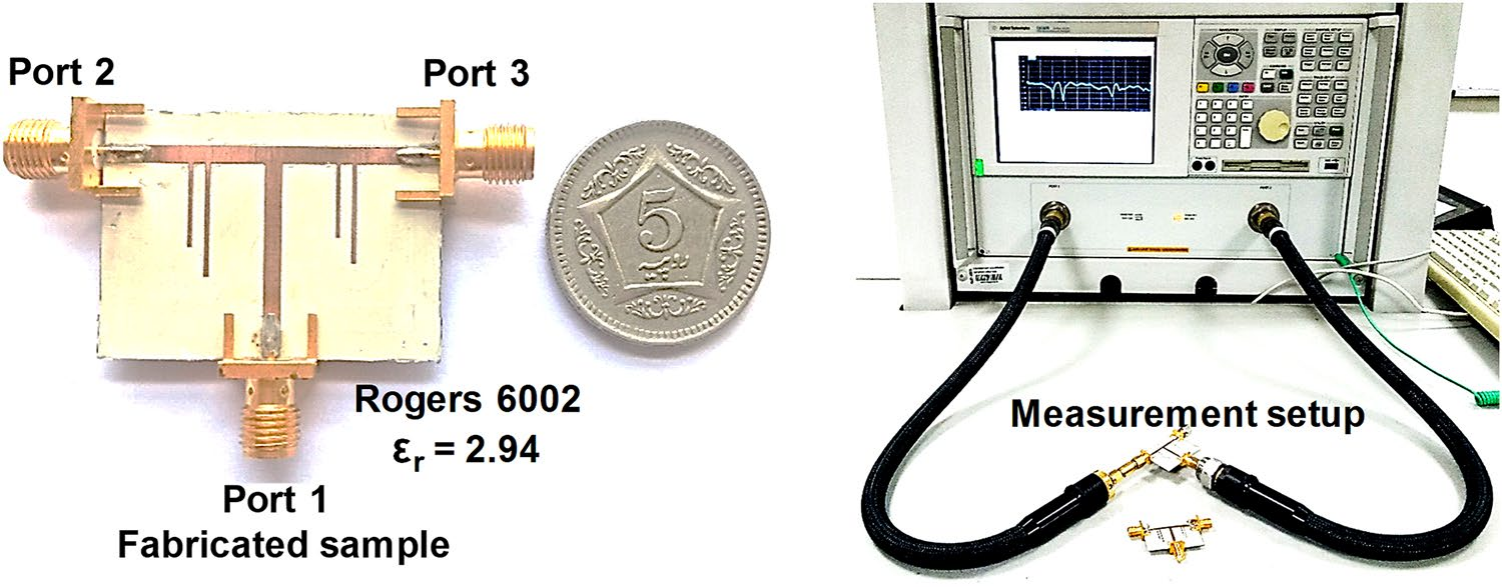
(Left) Fabricated sample of the proposed microstrip diplexer. (Right) Measurement setup showing the VNA connected to the fabricated device.

In this section, we present the practical realization of the diplexer (schematic prototype in Fig. 1b) which was simulated in the previous section with lossless parameters. Before the fabrication stage, the design was simulated in Agilent Momentum with realistic parameters for accurate results. To incorporate the dielectric and conductor losses, the Rogers 6002 substrate with a loss tangent of 0.0012 and the microstrip lines of conductor thickness 35 μm and conductivity of 5.8** × ** 10^7^ S/m are used. The diplexer is fabricated using the LPKF PCB prototyping machine. A prototype of the fabricated diplexer is shown in Fig. 4 has a compact size of 3 cm × 2.2 cm.

**Figure 5:**
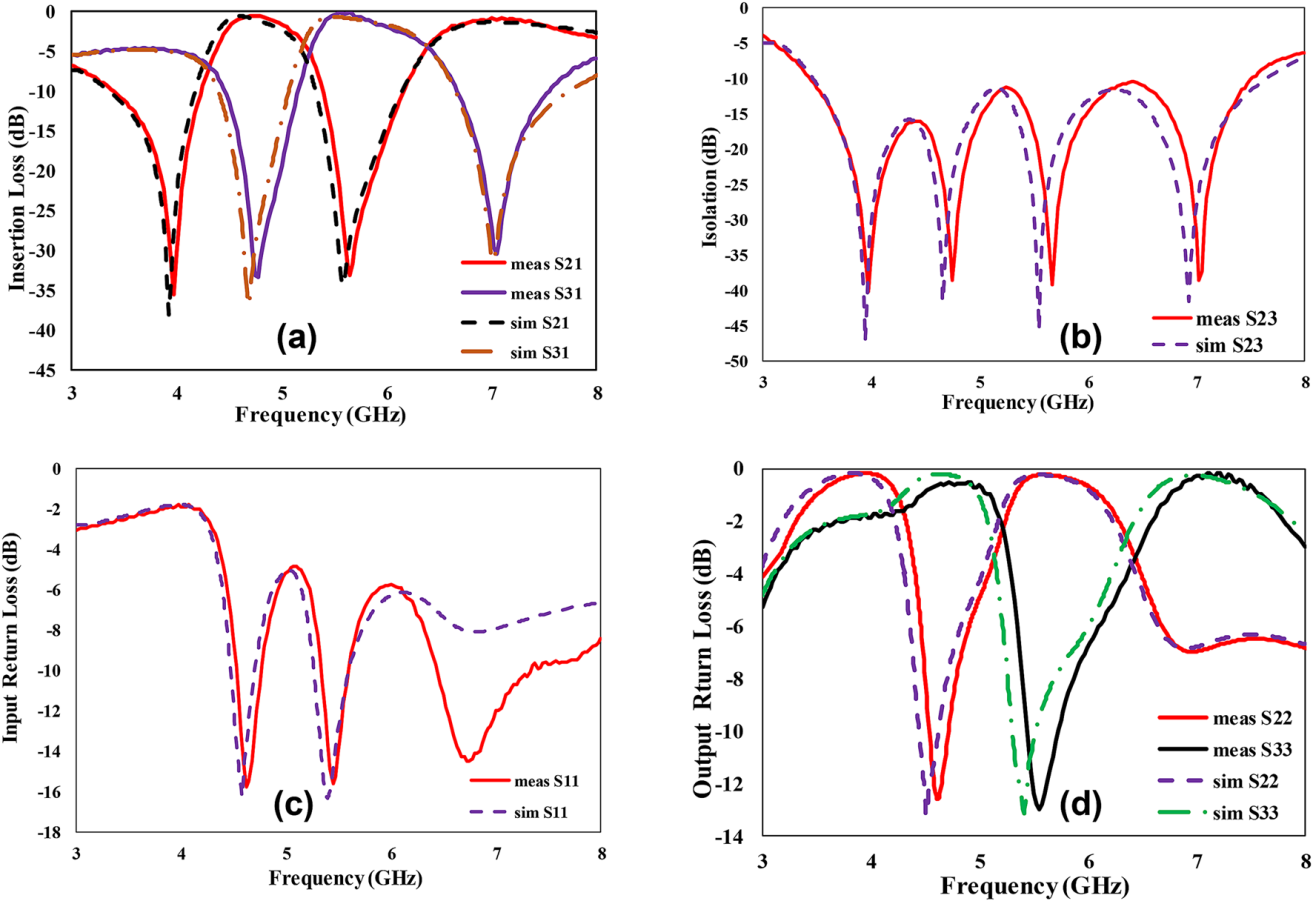
Scattering parameters of the proposed EIT-based microstrip diplexer (**a**) measured and simulated insertion loss, (**b**) measured and simulated isolation between the two channels, (**c**) measured and simulated input return loss (**d**) measured and simulated output return loss.

The transmission characteristics are measured by the E83628 vector network analyzer. As depicted in Fig. 5a, the insertion loss is 0.59 dB for the lower band and 0.61 dB for the upper band. The isolation of about 40 dB is achieved between the two output channels, as shown in Fig. 5b. The input return loss is better than − 15 dB at the designed frequencies, while the output return losses are well below − 12 dB as shown in Fig. 5c,d respectively. The fractional bandwidth is 16% for the lower passband and 18% for the upper passband. The measured and simulated results show very good agreement. The fabricated diplexer discriminates the low and high-frequency channels with the center frequencies of about 4.6 and 5.5 GHz. Small frequency shifts are due to the permittivity and fabrication tolerances. The transmission zeros are well placed and are located at 4 and 5.5 GHz for the lower frequency band, whereas 4.6 and 7 GHz for the upper-frequency band.

The response of the proposed diplexer is better as compared to recently proposed diplexers in terms of insertion loss and isolation. Moreover, the ease of reproducibility and scalability in the frequency of our proposed design is captivating. The proposed diplexer is compared in Table 1 with some already proposed microstrip based diplexers. The comparison shows that the proposed EIT-based technique in diplexer design can provide much better insertion loss along with much better isolation level.

**Table 1:** Comparison of the proposed diplexer based on the EIT-like effect with reported state-of-the-art diplexers.

References	Lower band/upper band (GHz)	S_21_/S_31_ (dB)	Isolation (dB)
Yan et al.^50^	3/4	1.5/1.1	> 20
Chen et al.^51^	1/1.2	2.24/2.22	> 37
Zhang et al.^52^	1.75/2.35	1.34/1.44	> 25
Chen et al.^53^	1.5/1.76	2.8/3.2	> 30
Yan et al.^54^	1.95/2.14	1.2/1.5	> 35
Guan^55^	1.82/2.41	2.2/2.1	> 30
This work	4.7/5.6	0.59/0.61	40

**Figure 6:**
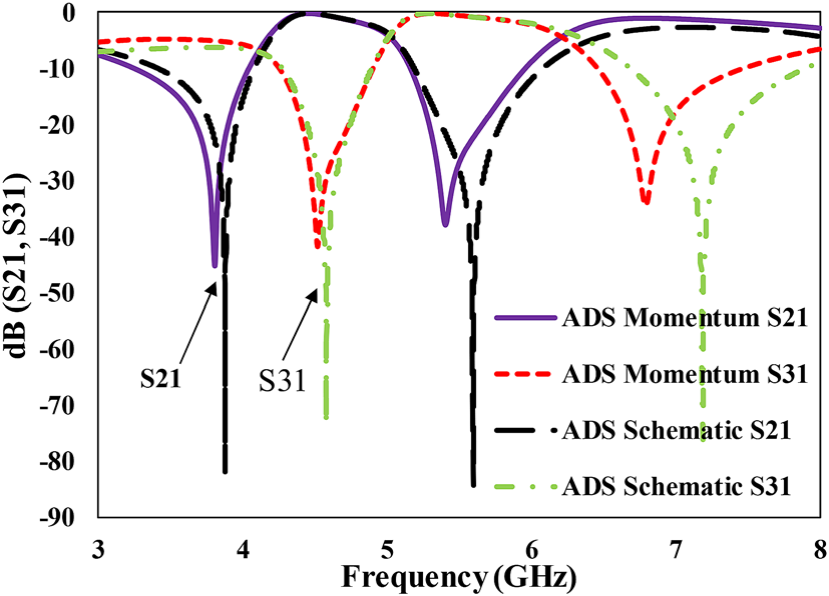
Demonstration of the comparison between ADS schematic and electromagnetic momentum simulations revealing that interference between the stubs is the responsible effect for the EIT-like effect as compared to coupling.

It would be instructive to comment on the difference between the mutual (inter-stub) interference effect (leading to the EIT-like effect) and the mutual coupling which results due to mutual inductance or capacitance. Reconsidering Fig. 1d and given the fact that the EIT-like transmission requires a perfect cancellation of currents due to destructive interference between the two stubs, a numerical investigation can be done to reveal the dominance of interference effect as compared to the coupling effect. Amin et al. demonstrated a detailed analysis of this cancellation phenomenon^45^, where it was shown that the two branches have oppositely polarized currents at the EIT-like transmission. Here we provide a comparison between the full-wave simulations and the circuit simulations using Agilent’s ADS. The circuit simulation treats the open-circuit stubs as independent lines instead of coupled lines, even then similar magnitudes of transmission coefficients in the passbands show that there is a negligible effect of the mutual coupling in the proposed design as depicted in Fig. 6. A similar conclusion was reported in an earlier work^56^, where the spectral separation between the resonance frequencies was not enhanced by coupling between the two step-impedance resonators when they were placed in on the same side of the main transmission line.

**Figure 7:**
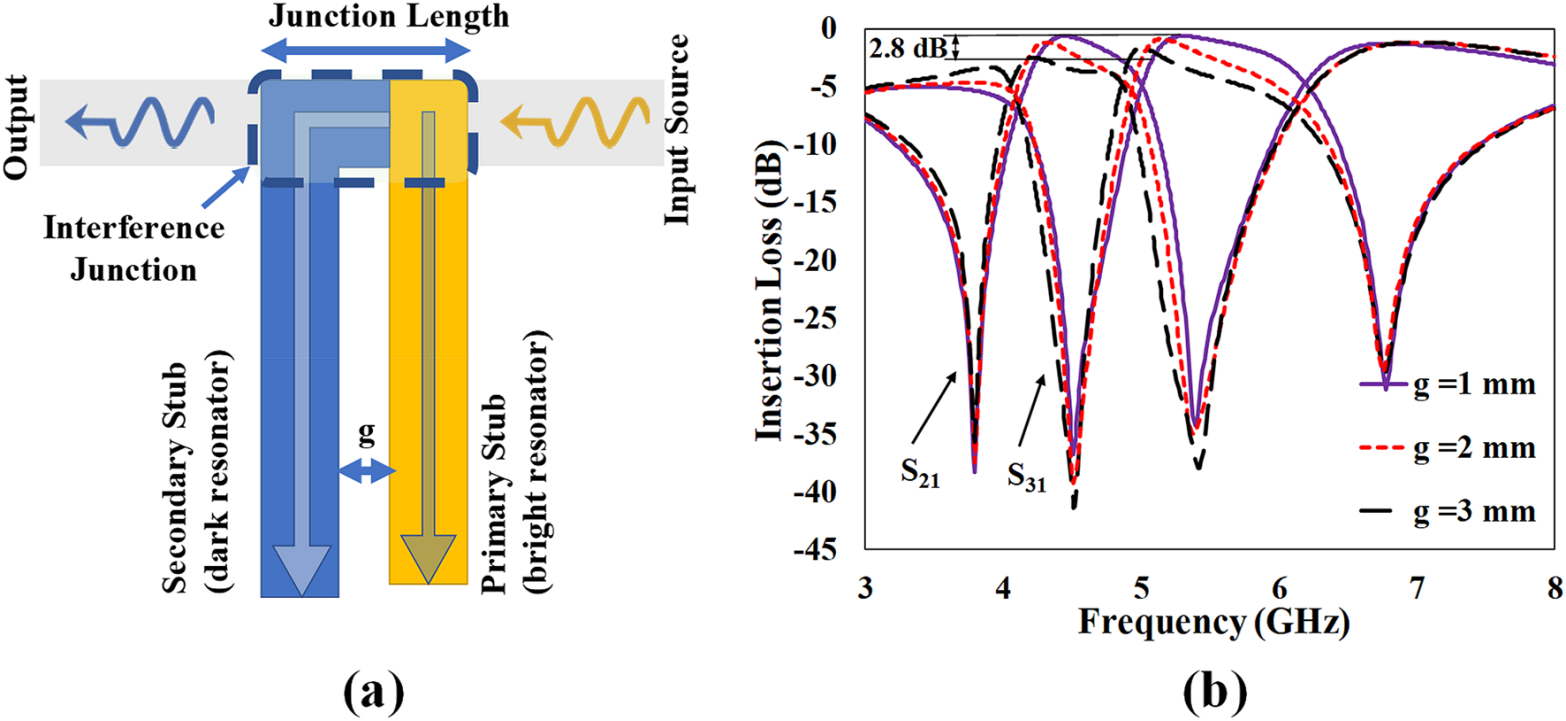
Effect of changing the junction length between the stubs (**a**) a representative example to show the junction length with dual stubs (**b**) The EIT-like response of full diplexer, the increase in the insertion loss of 2.8 dB is observed due to increase in the inter-resonator distance from 1 to 3 mm.

In order to investigate the effect of junction length (the gap between the pair of stubs), the gap is increased from 1 to 3 mm. As depicted in Fig. 7, the increase in the inter-resonator distance (or the junction length) results in a lossy and distorted EIT-like transmission causing a 2.8 dB increase in the insertion loss. However, the location of the transmission zeroes is negligibly affected. Therefore, it can be safely inferred that the destructive interference dominates the EIT-like transmission and that the EIT-like effect is enhanced when the inter-stub separation is reduced. However, the spectral separation between the resonance frequencies is not perturbed by the coupling effect in this topology.

## Discussion

The recent breakthroughs in the CMOS technology have made the on-chip integrated antenna a possibility^57,58^, thus paving the way to “true” fully integrated CMOS transceivers. The only missing component to a full “single-chip RF transceiver integration” is an on-chip diplexer. With the involvement of active components and complex components such as on-chip transformers, such designs were marked by restricted power consumption. On the other hand, the proposed diplexer is a combination of transmission line segments and open-circuit stubs. Therefore, the structure is simple to implement in any technology and supports sharp filtering actions. A vast majority of contemporary diplexers are based on the surface acoustic wave (SAW) that cannot be integrated on a CMOS chip. The on-chip solution consists of higher order inductor-capacitor based filters, which if built on-chip, occupy much expensive silicon area. Furthermore, their high tolerances (~ 10 to 20%) make them tougher to design them in a high frequency regime.

**Figure 8:**
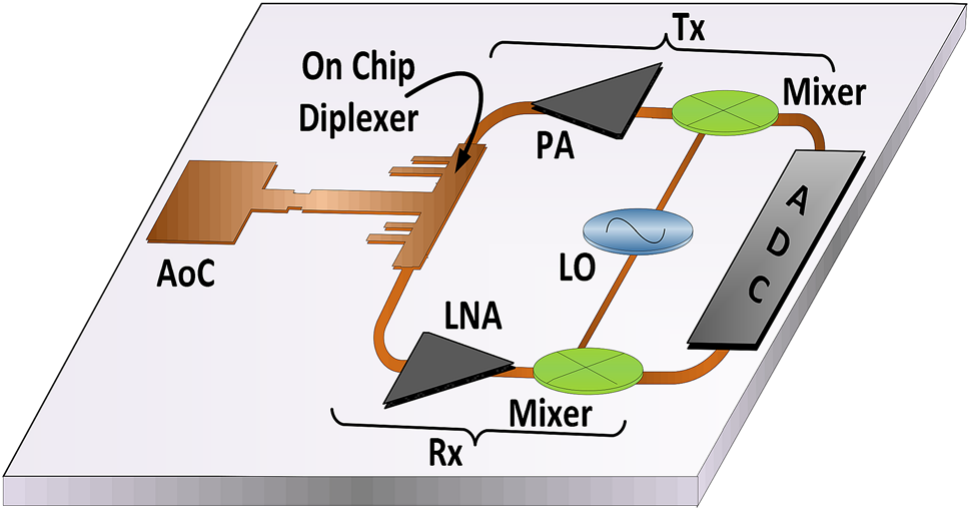
The block diagram of one tile of the future mm-wave on-chip MIMO CMOS transceiver with integrated on-chip antenna (AoC) and proposed diplexer circuit for frequency division duplex (FDD) systems. The explanation of the abbreviations used in the figure is as follows. PA = power amplifier, LNA = low noise amplifier, ADC = analog to digital converter.

To solve some of these shortcomings, the idea of the proposed integrated diplexer based on the EIT-like wave discrimination is illustrated in Fig. 8. The proposed microwave device can be used to discriminate the electromagnetic waves that are coming towards it from the on-chip antenna and the transceiver module. The designed device is highly compact, simple to implement and scalable to many desired frequency bands. The design based on the EIT-like effect has a potential to be fully integrated (if scaled up to high frequencies as shown in Table 2) with other on-chip components like an on-chip antenna, low noise amplifiers (LNA), power amplifiers (PA) and mixers for millimeter-wave 5G multiple-input multiple-output (MIMO) transceiver. These futuristic MIMO based systems will have multiple transceiver chains including on-chip antennas for the beamforming. One tile of such future systems is shown in Fig. 8. We expect that following the proposed design methodology, low-cost fully integrated RF transceivers can be commercially produced with potential applications in automotive radars, beamforming MIMO transceivers, phased array systems, WPAN networks and mm-wave 5G transceivers^59–66^.

From the integrated circuit perspective, it is worth mentioning that the dual-stub circuit can be designed in a more compact way by either mitering, meandering or by utilizing more than one metal layer of the CMOS IC stack. The dimensions of the diplexer scaled to the higher frequencies of 28 to 32 GHz for mm-wave 5G systems and 60, 77 and 90 GHz are listed along with those of the C-band design in Table 2. It can be observed by looking at the lengths of open-circuit stubs that the diplexer can be practically scalable on a single CMOS chip since the calculated scaled-up dimensions are quite compatible to the contemporary designs presented in some of the recent studies^60,64–66^.

**Table 2:** Approximate lengths for low and high-frequency diplexer realization.

Frequency (GHz)	Stub length (mm)	Dielectric constant
4	12.16	2.94
4.7	10.35	2.94
5.6	8.68	2.94
7	7	2.94
28	1.29	4.3
32	1.13	4.3
60	0.61	4.3
77	0.47	4.3
90	0.42	4.3

## Conclusion

We exploit the EIT-like interference effect that demonstrates a sharp transmission response and strong dispersion characteristics in the microwave spectrum. The idea of interference-based devices is inspired by the quantum mechanics phenomenon of EIT which creates passbands in the otherwise opaque medium through laser interference. Since the filtering action is obtained by associated steep phase profiles, single-stage EIT-based structures can discriminate closely located frequencies with high isolation. We presented a compact three-port C-band microstrip diplexer with a simple design that consists of pairs of two unequal open-circuit stubs towards the path of each output port which interfere destructively to form the EIT-like passbands for diplexing action. The insertion loss of the lower and upper passbands is 0.59 dB and 0.61 dB respectively, with good isolation of about 40 dB. Simulations and measured results show quite good agreement. The comparison with other reported diplexers shows that the performance of the proposed diplexer based on the EIT-like effect is much better in terms of insertion loss, isolation and simplicity of the design. The proposed diplexer has a simple geometry that consists of all passive transmission lines, hence it is readily scalable to desirable frequencies. Moreover, we also suggest a future work for on-chip diplexer (if scaled-up to high frequency) that can be potentially implemented in CMOS-based transceivers. With the realization of an on-chip diplexer, an all-integrated transceiver for radar and high power applications is possible.
